# Oral health interventions for people living with mental disorders: protocol for a realist systematic review

**DOI:** 10.1186/s13033-020-00357-8

**Published:** 2020-03-24

**Authors:** Amanda Kenny, Virginia Dickson-Swift, Mark Gussy, Susan Kidd, Dianne Cox, Mohd Masood, David Azul, Carina Chan, Bradley Christian, Jacqui Theobold, Brad Hodge, Ron Knevel, Carol McKinstry, Danielle Couch, Nerida Hyett, Prabhakar Veginadu, Nastaran Doroud

**Affiliations:** 1 Violet Vines Marshman Centre for Rural Health Research, LaTrobe Rural Health School, P.O. Box 199, Bendigo, VIC 3552 Australia; 2 College of Social Science University of Lincoln, Brayford Pool, Lincoln, Lincolnshire LN6 7TS UK; 3 Mental Health Nursing, Mental Health Nurse Practitioner, Victoria University, Footscray, VIC Australia; 4 LaTrobe Rural Health School, Health School, Dentistry and Oral Health, P.O. Box 199, Bendigo, VIC 3552 Australia; 5 School of Psychology and Public Health, LaTrobe University, Bendigo, Australia

**Keywords:** Mental disorder, Oral health, Realist systematic review

## Abstract

**Background:**

The increasing number of people who experience mental disorders is a global problem. People with mental disorders have high rates of co-morbidity and significantly poorer oral health outcomes than the general public. However, their oral health remains largely a hidden and neglected issue. A complex range of factors impact the oral health of this group. These include anxiety and dental phobia, dietary habits, including the heavy consumption of sugary drinks, substance misuse of tobacco, alcohol, and/or psychostimulants, the adverse orofacial side effects of anti-psychotic and anti-depression medications, and financial, geographic, and social barriers to accessing oral health care.

**Methods:**

The aim of this realist systematic review is to (a) identify and synthesise evidence that explores oral health interventions for people living with mental disorders; (b) explore the context and mechanisms that have contributed to the success of interventions or the barriers and challenges; (c) produce program theories on causal, contextual and mechanistic factors to facilitate outcomes and (d) produce recommendations and guidelines to guide future oral health interventions for people with mental disorders at both the policy and practice level. Using a five-step process, that incorporates primary data collection from key stakeholders, a beginning theoretical framework will be developed to describe contextual and mechanistic factors and how they might impact on the success or failure of oral health interventions for people with mental disorders. Key database searches will be conducted, with data extraction focused on the factors that might have impacted on intervention implementation and outcomes. Quality appraisal of studies will occur, and the theoretical framework will be populated with extracted data. Stakeholder input will support the development and refinement of a theory on oral health interventions for people with mental disorders.

**Discussion:**

This will be the first review to take a realist approach to explore the broad scope of causal factors that impact on the success or failure of oral health interventions for people with mental disorders. The approach includes extensive stakeholder engagement and will advance realist systematic review methodology. Review outcomes will be important in guiding policy and practice to ensure oral health interventions better meet the needs of people with mental disorders.

*Systematic review registration* This review protocol is registered with PROSPERO (Number) 155969.

## Introduction/background

The aim of this realist systematic review [1–3] is to identify and synthesise studies that explore oral health interventions for people living with mental disorders. The terms mental disorder and mental illness are often used interchangeably. In this review, we use the term mental disorder consistent with the language of the World Health Organization (WHO) [4].

Mental disorders describe a spectrum of conditions affecting people’s thinking, behaviour, and relationships [5]. It is estimated that almost 50% of people will experience some form of mental disorder in their lifetime [6, 7]. Authors argue that mental disorders are underreported across most countries. In the United States (US), anxiety and depression impact 11.48% of the total population, in the United Kingdom (UK) 8.77%, Australia 11.2%, and in countries such as China rates are reported as 6.34%. Across the world, depression and anxiety disorders are estimated to cost US$1 trillion per year [8]. For disorders such as schizophrenia and bipolar, prevalence rates are: US 0.98%, UK 1.35%, Australia 1.5%, and China 0.66% [9].

Mental disorders are among the leading causes of disability, accounting for 7.4% of global disability-adjusted life years (DALYs), and 22.7% of global years lived with disability YLDs [10]. The severity of mental disorders varies and can lead to persistent episodic symptoms that impact functioning, with associated requirements for long-term care [6].

People diagnosed with mental disorders experience high rates of co-morbidity [11]. Life expectancy of people diagnosed with low prevalence mental disorders, such as schizophrenia, schizoaffective disorder, bipolar disorder, and delusional disorders [12] is between 10 and 20 years lower than the general population [11].

Good oral health is integral to general health and quality of life and is a fundamental human right [13–15]. However, approximately 3.5 billion people live with untreated oral conditions [16, 17]. Lives are negatively impacted, and millions of productive hours are lost annually as a result of poor oral health [13, 15, 18–20]. People with mental disorders have significantly poorer oral health outcomes than the general population [21–23]. Kisely [21] refers to a bi-directional association between oral health and mental health. Actual and anticipated dental treatment can lead to anxiety and dental phobia. Many mental health disorders (for example, psychotic and eating disorders) are associated with higher prevalence and greater severity of dental disease, including erosion, caries, and periodontitis [21]. People hospitalised for their mental disorders have the worst oral health outcomes [24–26]. There is a lack of current studies that have explored general anaesthesia associated with dental treatment in people with mental disorders. However, in the broader category of special needs, authors have highlighted increased demand for general anaesthesia associated with dental treatment and much higher anaesthetic risk associated with multiple comorbidities [27–30]. Exploration of oral health intervention studies that consider general anaesthesia is a gap.

People experiencing mental disorders are negatively impacted by many social determinants of health including poverty, unemployment, housing insecurity, and social isolation [31, 32]. These issues are also significant risk factors/indicators for poor oral health. Poor oral health in people with mental disorders is associated with: poor dietary habits and poor nutrition, heavy consumption of sugary drinks; comorbid substance misuse of tobacco, alcohol, and/or psychostimulants and other medications; and financial, geographic, and social barriers to accessing oral healthcare [21]. People with severe mental disorders are more susceptible to oral disease because of poor oral hygiene [26], dental phobia [27, 28], dental costs [29, 30], difficulty in accessing health care facilities [30], and the adverse orofacial side effects (including bruxism and xerostomia) of anti-psychotic and anti-depressant medications [22, 33–35].

Poor oral health can contribute further to the social withdrawal, isolation, and low self-esteem of those with mental disorders who are already highly vulnerable [23, 36, 37]. There is a close association between dental disease, coronary health disease, stroke, diabetes, and respiratory disease [38–42], conditions that are commonly experienced by people with mental disorders [22, 40]. For this group, poor oral health is a critical issue but is often ignored by policymakers and service providers [21, 36].

Previous systematic reviews have explored the oral health of people with schizophrenia and bipolar disorder [22, 36, 43, 44]. A meta-analysis by Matevosyan [36] examined the prevalence of suboptimal oral health in adults with severe mental illness, including poor oral hygiene, increased intake of carbonates, poor perception of oral health self-needs, duration of psychotropic treatment, and reduced access to dental care. Two consecutive systematic reviews and meta-analyses investigated the association between edentulism (missing teeth) and measures of dental caries [decayed, missing, filled teeth (DMFT) or surfaces (DMFS)], and serious mental disorders [22, 44]. The findings suggest that people with serious mental disorders face greater likelihood (2.8 times) of losing all their teeth and significantly higher Decayed, Missing and Filled Teeth (DMFT) and Decayed Missing and Filled Surfaces (DMFS) scores compared to the general population. One review focused on the effects of oral health education, motivational interviewing, monitoring, and standard care on oral health and quality of life for people with serious mental illness [43]. The authors concluded that there was insufficient evidence from the studies to recommend an intervention.

Reviews have been conducted on the oral health of people diagnosed with eating disorders [45] and demonstrated significantly higher risk of dental erosion caused by vomiting when compared to the general population and significant association between dental caries and dry mouth. Other systematic reviews by Kisely et al. [22], Cademartori et al. [46] and Baghaie et al. [47], have identified a greater burden of dental caries and periodontal disease in populations with anxiety and depression and substance abuse disorders.

Authors [22, 36, 43, 44] have highlighted a lack of evidence on the effectiveness of oral health interventions for people with mental disorders. There have been calls for the training of mental health professionals and closer collaboration between all health professionals [48, 49]. Recommendations for further research include studies focused on oral health education and promotion within mental health service settings (including inpatient and community settings) [23, 49]. While there are no recent studies that have explored dental treatment [27] and general anaesthetics for people with mental illness, authors have called for preventive dental programs for vulnerable populations as a means to reduce anaesthetic risks [30]. A recent review by Slack-Smith and colleagues reported that barriers to good oral health for people with mental disorders fell into three categories; individual, organizational (including health providers), and system-level [23]. While these reviews are useful, no authors have produced a comprehensive synthesis of the context and mechanisms that influence oral health interventions for people with mental disorders. There is a lack of evidence-based theory to guide policy and practice. This review addresses this gap.

In this review, we conceptualise poor oral health as a ‘wicked problem’, one that has a significant impact but has proven to be intractable [50–52]. Wicked problems are resistant to usual problem-solving approaches, require action by a diversity of stakeholders, require major behaviour change at system, service and individual levels, and most ‘wicked problems’ are characteristic of chronic policy failure [50]. Authors agree that poor oral health for people with mental disorders must be addressed at a systems level [23, 53, 54], rather than a reliance on more traditional approaches where the individual and the context are reduced to independent, quantifiable factors [51, 54]. By grounding this review in critical realism [1–3], we will extend beyond previous systematic reviews and undertake an in-depth exploration at the individual, service, and system levels, to unravel the impact of what works for whom, in what context and how [55]. This will enable an exploration of the success and failures of interventions and the many combinations of the two. This contextually bound approach to causality is represented as context + mechanism = outcome [1, 2].

Abayneh, Lempp, Manthorpe and Hanlon [56] draw together literature to define key realist concepts and terms. Context is defined as a configuration of factors that are not always directly connected to an intervention. These could include features of the intervention site and its location, human resources and the way they interact, and culture. Mechanisms are defined as a ‘generative force triggered in particular contexts’ or cognitive or emotional responses of individuals experiencing an intervention; carers, service staff, community members, and those in the broader health system. They state that consideration of mechanisms is essential in moving beyond what happened, to why, for whom, and in what circumstances. The interaction between the context and the mechanism, or how people respond, can be based on factors such as beliefs, values, preferences, and thought processes. The resultant outcomes might lead to short, medium- or longer-term change and can be intended or unintended [56].

We hypothesise that the contexts in which oral health interventions are delivered to people with mental health disorders are multi-faceted and dynamic and that interventions rarely work in the same way within different contexts. Realist systematic reviews are interpretive, and theory driven. Traditional systematic reviews have focused on intervention or program effectiveness. However, in most cases, there is little indication of how the program or intervention worked, what contributed to the success, or the barriers and challenges in implementation. Few reviews explore how the context, circumstances and stakeholders influence outcomes [1]. Realist systematic reviews explore the interconnectedness between context, mechanism, and outcomes (CMO) [55, 57, 58]. From this review, we will develop extensive understandings about oral health interventions for people with mental disorders. The developed theory will guide policy and practice.

### Aim and review questions

The aim of this realist systematic review is to (a) identify and synthesise studies that explore oral health interventions for people living with mental disorders; (b) explore the context and mechanisms that have contributed to the success of interventions or the barriers and challenges; (c) produce program theories on causal contextual and mechanistic factors to facilitate outcomes and (d) produce recommendations and guidelines to guide future oral health interventions for people with mental disorders at both the policy and practice level [57]. The DSM-5 Diagnostic Classification has been used to guide the disorders that will be considered [59]. The following review questions will be answered:

1. What are the contextual factors at the local, service, and system level that impact on the success or failure of oral health interventions for people with mental disorders?
2. What are the mechanisms that have led to success or failure?
3. Are there contextual and mechanistic factors that are consistent across studies of oral health interventions for people with mental disorders?
4. What causal theories can describe the impact of these contextual and mechanistic factors, and how might they influence policy and practice?

This review protocol is registered with PROSPERO (Number) 155969.

## Methods

The methods used in this review are novel but aligned to the theory-driven approach that underpins the realist systematic review method [56, 60]. While the approach is based on the five-step process of Pawson et al. [1]: clarifying scope, searching for evidence, appraising primary studies and extracting data, synthesising evidence and drawing conclusions, and disseminating, implementing and evaluating, we strengthen the review through integrated primary data collection. A key feature of realist systematic reviews is the input of stakeholders throughout the review to support theory generation, knowledge translation, and impact [1, 58]. Cooper and colleagues [61] used this combination of primary (stakeholder input) and secondary (literature searching and synthesis) data in their review of complex interventions to prevent adolescents from engaging in multiple risk behaviours. They argued that the incorporation of primary data in their review gave greater insights into causal factors that might not be identified within the literature and, importantly, provided opportunities for adolescents to have a strong voice in theory development. In this review, we will draw on our extensive experience of working with policymakers, commissioners, service providers, and people with mental disorders [62–64] and our use of innovative methods of data collection, including the use of blogs [65]. Internationally, health policymakers confirm the need for greater public participation in research [66] and mental health consumers in all stages of service design, implementation, and evaluation [62, 67, 68]. The approach will be multifaceted, flexible, and iterative and will involve triangulation of findings across the entire review.

### Clarifying scope

The review team is multidisciplinary (oral health and dentistry, nursing, public health, psychology, sociology, mental health, social work, and allied health) to capture a multitude of perspectives in the initial development of the review. Pawson and colleagues [1, 58] confirm the need to ‘scavenge’ ideas in this phase to develop an initial theoretical framework. An initial search of the literature will be undertaken to map out beginning theories of how and why oral health interventions for people with mental disorders might work. We will take a local and global approach to stakeholder involvement. A state-wide, Australian stakeholder forum (policymakers, commissioners, service providers, consumer peak bodies, mental health consumers, carers and other interested parties) will be held to consider our initial scoping work and provide expert input into a beginning theoretical framework. This framework will describe contextual and mechanistic factors that might impact on the success or failure of oral health interventions for people with mental disorders. We will advertise this forum widely through existing professional and consumer networks, print, and social media. To facilitate nationally and international input, an open-access blog will be used to house the beginning theoretical framework, and we will drive input into this framework via social media. The blog will link to a website where findings will be regularly updated. Broad input will be an important component of our integrated knowledge translation approach. Integrated knowledge translation is defined as a process of engagement between researchers and knowledge users (those who will make use of research findings to inform decisions) [69, 70]. This approach to knowledge translation supports rapid societal impact, a key direction in international research policy [71, 72].

### Phase two searching for evidence

Using the expertise of the research team a number of key concepts to guide the search were developed. Table 1 outlines the key concepts that will be used in the search.

**Table 1:** Key concepts for search

Concept 1: oral health	Concept 2: mental health disorders	Concept 3: interventions
Oral health	Psychotic disorders	Program development
Oral hygiene	Anxiety disorder	Health promotion
Dental caries	Eating disorder	Program evaluation
Dental care	Depressi*	Health program
Dental health	Mental disorders	Health intervention*
Dental hygiene	Schizophrenia	Oral health education
Oral care	Bipolar disorder	Oral health program
Periodontal disease	Mental illness	Oral health promotion
Periodontitis		Health system
Tooth loss		Health initiative
Edentulism		
Xerostomia		

With the support of a specialist healthcare librarian, detailed search strategies will be developed for each database [Medline Ovid, Embase Ovid, PsycINFO, Academic Search Complete, CINAHL EBSCO, Cochrane Oral Health Trials Register, Cochrane Central Register of Controlled Trials (CENTRAL) based on the one developed for MEDLINE (Ovid) [see Table 2]. MeSH terms will guide the search. Search terms will include truncation or keywords, the use of thesaurus terms and subject headings, and combining terms and search strings with the appropriate Boolean operators.

**Table 2:** Example search for medline

Search#	Concepts
S1	[Oral health or oral hygiene or dental caries or dental care or dental health or dental hygiene or oral care or periodontal disease or periodontitis or tooth loss or edentulism or xerostomia).mp. [mp = title, abstract, original title, name of substance word, subject heading word, floating sub-heading word, keyword heading word, organism supplementary concept word, protocol supplementary concept word, rare disease supplementary concept word, unique identifier, synonyms]
S2	MH oral health/
S3	[Psychotic disorder* or Anxiety disorder* or Eating disorder* or Depressi* or Mental disorder* or Schizophrenia or Bipolar disorder* or Mental illness*).mp. [mp = title, abstract, original title, name of substance word, subject heading word, floating sub-heading word, keyword heading word, organism supplementary concept word, protocol supplementary concept word, rare disease supplementary concept word, unique identifier, synonyms]
S4	MH mental disorders/
S5	[Program development or health promotion or program evaluation or health program or health intervention*OR oral health education OR oral health program or Oral health promotion or health system or Health initiative).mp. [mp = title, abstract, original title, name of substance word, subject heading word, floating sub-heading word, keyword heading word, organism supplementary concept word, protocol supplementary concept word, rare disease supplementary concept word, unique identifier, synonyms]
S6	MH Oral Hygiene/or Health Education, Dental/or Health Promotion/or oral health promotion.mp
S7	1 OR 2
S8	3 OR 4
S9	5 OR 6
S10	7 AND 8 AND 9
S11	limit 10 to English language

#### Study designs

In line with the purpose of a realist systematic review [73], quantitative, qualitative, and mixed-method studies will be included. There are no data range limitations. Included studies must be published in English, reflecting the significant resource implications associated with translation [74].

#### Participants and setting

Reviewed studies can include participants diagnosed with any mental health disorder. Studies can be carried out in any setting (including inpatient and community settings) and can be in any geographical location.

#### Interventions

The review will include any interventions designed to address oral health outcomes in people with mental disorders. Content of the interventions could include some or all of the following: dental and oral health, oral disease and impact on health, general anaesthesia associated with dental treatment, dietary interventions related to improving oral health, oral hygiene measures, best oral health practices for people with mental health disorders, oral hygiene promotion and skills training (for people with mental health disorders or those who care for them in both inpatient and community settings).

#### Screening of studies

The screening process will be conducted in four phases: (1) title and abstract, (2) full text, (3) search of the reference lists and (4) search of citations of all included studies for any further suitable studies. This phased approach aims to capture a breadth of studies.

Table 3 outlines the inclusion and exclusion criteria that will guide the review.

**Table 3:** Inclusion and exclusion criteria

	Inclusion	Exclusion
Participants	Participants diagnosed with any mental health disorder	Anything outside the inclusion criteria
Setting	Any setting (including inpatient and community settings) and can be in any geographical location	None
Interventions	Any intervention designed to address oral health outcomes in people with mental disorders	Anything outside the inclusion criteria
Study design	All study designs	None
Publication type	Peer-reviewed publications of original research	Non peer reviewed publications, editorials and opinion pieces; conference presentations and/or abstracts; commentaries
Outcomes	Oral health and related outcomes such as oral health knowledge or oral health behaviours	Anything outside the inclusion criteria
Language	English	Non-english
Date	All date range	None

Endnote (bibliographic software program) and Covidence (Cochrane’s systematic review management software) will be used to manage search results. A sample of 25 articles will be assessed by all reviewers to ensure reliability in the application of the inclusion and exclusion criteria. Discussion will occur to ensure that the team are applying criteria in the same way. Covidence software supports a blind review process, and at each phase, at least two reviewers will screen articles. Conflicts are highlighted by the software and discrepancies will be discussed until consensus is reached. To ensure inclusion of all relevant studies, the reference lists of all studies captured as a result of phases 1 and 2 will be examined manually, and Web of Science/Scopus will be used to identify citations of all included full-text articles. The Preferred Reporting Items for Systematic Reviews and Meta-Analyses, (PRISMA) [75] checklist will be used to guide the review, and all stages of the study selection will be documented using a PRISMA flow chart [75].

#### Data extraction

To address the review questions, data will be extracted on population, study design, intervention, and outcomes. Consistent with realist review methods and the research questions, data extraction will include the contextual factors at local through to system level, that impact on the success or failure of oral health interventions for people with mental disorders and the mechanisms that have led to success or failure. A minimum of two reviewers will check data extraction tables, and disagreements will be discussed until consensus is reached. As data is extracted, the beginning theoretical framework from phase one will be populated with evidence and shared, using the website and blog.

#### Quality appraisal

As the aim of realist systematic reviews is to identify the interplay between context, mechanism, and outcome [56], no studies will be excluded based on methodological quality. Three tools will be utilised to assess studies depending on study design: the Cochrane Collaboration Tool for Assessing Risk of Bias in Randomised Trials [76] and the Risk of Bias in Non-Randomised Studies of Interventions (ROBINS-I) [77] for quantitative studies and the Critical Appraisal Skills Programme (CASP) Checklist for Qualitative Research [78]. A minimum of two reviewers will assess all studies and disagreements between authors will be resolved through team discussion. Quality appraisal results will be presented in a single table.

#### Data analysis

Data synthesis in realist reviews is guided by the RAMESES Standards [2, 79], which comprises a combination of inductive and deductive analytical processes directed at further building an explanatory theory about the context, mechanism, outcome (CMO) relationships of the interventions under investigation. Two reviewers will independently code data segments representing the CMO in each reviewed article. The research team will produce a narrative synthesis that draws upon Pawson’s [55] techniques: ‘juxtaposing’ sources to enable broader insights, ‘reconciling’ different outcomes within different contexts, ‘adjudicating’ between studies on the basis of methodological strengths and weaknesses, ‘consolidation’ of explanations of differences between studies, and ‘situating’ studies in their contexts. The overall aim will be to identify contextual and mechanistic factors that are consistent across studies of oral health interventions for people with mental disorders.

### Dissemination, implementation, and evaluation

An additional state-wide, stakeholder forum will be conducted to refine and confirm the causal theory on the impact of contextual and mechanistic factors. Discussion will occur on how the theory might influence policy and further development and implementation of interventions. As in the first phase stage, the findings from this forum will be shared via the website and blog for further input.

An integrated knowledge translation (iKT) [69, 70] approach will be adopted throughout this review. To increase the relevance, applicability and impact of the review, key stakeholder participation will be widely utilized. In addition to traditional academic methods of dissemination such as publications and conference presentations, other communication modes will be used, including infographics, blogs, social media postings, webinars, and podcasts.

### Strengths and challenges

Previous systematic reviews on the oral health of people with mental disorders have focused mainly on oral health risks, barriers for oral health, and the effectiveness of interventions. Taking a realist review approach will add significantly to the knowledge base as context and mechanism will be considered. The work of Abayneh et al. [56], provides a good guide to differentiating between context and mechanism, and we will draw on their processes to ensure team consistency in how key terms and concepts are applied. Team discussion and codebooks will be used to document decisions. We acknowledge the challenges of reproducing a realistic systematic review because of the approaches taken [80]. By detailing each step and documenting and tabulating summary tables of what is found, we will clearly indicate how conclusions were made.

## Discussion and conclusion

This will be the first review to take a realist approach to explore the contextual and mechanistic factors from individual, service, and system-level that impact on the success or failure of oral health interventions for people with mental disorders. We will identify factors that are consistent across studies to develop a theory on how the design and implementation of oral health interventions might better meet the needs of the rising number of people with mental disorders.

The novel approach to active stakeholder engagement advances realist systematic review methodology. Through extensive local, national, and international stakeholder engagement, we will gain greater insights into causal factors that might be missed with a more conventional systematic review. Engaging stakeholders in this early stage is also critical for future dissemination and implementation of evidence. Our approach will ensure that people with lived experience of mental disorders are provided with opportunities to inform the design and development of future oral health interventions.

## Data Availability

The datasets used and/or analysed during the current study are available from the corresponding author on reasonable request.

## Abbreviations

WHO: World Health Organization
DALYs: Disability-adjusted life years
YLDs: Years lived with disability
DMFT: Decayed, missing or filled teeth
DMFS: Decayed, missing or filled surfaces
CMO: Context, mechanism, outcome
PRISMA: Preferred reporting items for systematic reviews and meta-analyses
RAMESES: Realist and meta-narrative evidence syntheses: evolving standards
ROBINS-I: Risk of bias in non-randomised studies of interventions
CASP: Critical appraisal skills programme
iKT: Integrated knowledge translation
